# Protein Quantity and Source, Fasting-Mimicking Diets, and Longevity

**DOI:** 10.1093/advances/nmz079

**Published:** 2019-11-15

**Authors:** Sebastian Brandhorst, Valter D Longo

**Affiliations:** 1 Longevity Institute, School of Gerontology, and Department of Biological Sciences, University of Southern California, Los Angeles, CA, USA; 2 FIRC Institute of Molecular Oncology, Italian Foundation for Cancer Research Institute of Molecular Oncology, Milan, Italy

**Keywords:** protein intake, protein source, fasting-mimicking diet, healthspan, longevity

## Abstract

Dietary modifications, including caloric restriction, dietary restriction, various intervals of fasting, and even limiting the time when food is consumed can have a pronounced impact on longevity. In addition, dietary modifications are powerful interventions to delay, prevent, or treat many aging-related diseases such as cancer and diabetes. Restricting amino acid and protein intake generally decreases aging-related comorbidities and thereby increases health and longevity. However, chronic dietary interventions are likely not feasible for most people due to low adherence to dietary protocols or resistance to drastic changes to lifestyle, and might even cause detrimental effects, possibly by negatively affecting the immune system and wound healing. The periodic use of low-protein, low-calorie fasting-mimicking diets (FMDs) has the potential to promote health benefits, while minimizing the burden of chronic restriction. Protein restriction and FMDs together have the potential to play an important complementary role in medicine by promoting disease prevention and treatment, and by delaying the aging process at least in part by stimulating stem cell–based regeneration in periods of normal food intake after periodic FMD cycles. The aim of this narrative review is to summarize research on the impact of protein restriction on health and longevity in model organisms and to discuss the implementation of an FMD in mice and in human clinical trials and its effects on biomarkers of healthy aging. Taking into account the importance of sex on aging and diet, we include this information in all discussed studies. Whereas for some model organisms of aging, such as rodents, many studies are available, results are more limited for primates and/or humans.

## Introduction

The evolutionarily conserved role for nutrient-sensing pathways in health and longevity extends from simple organisms to rodents and nonhuman primates. In eukaryotic model organisms, the growth hormone/insulin-like growth factor-I (GH/IGF-I) pathway and its downstream effectors, target of rapamycin (TOR)/ribosomal protein S6 kinase (S6K) and the adenylate cyclase-protein kinase A (PKA) pathways, are involved in the regulation of metabolism and growth in response to nutrient abundance and thereby promote aging (1). Specific macronutrients activate these pathways and, notably, protein and amino acids play a central role in the metabolic response to affect growth and reproduction, healthspan, physiology, and longevity (2).

The effect of the GH/IGF-I pathway on longevity was first demonstrated in *Caenorhabditis elegans* (3, 4). Subsequent studies in multiple model organisms identified that orthologs of genes functioning in the TOR/S6K and RAS/cAMP/PKA signaling cascades also provided evidence for the conserved regulation of aging by progrowth nutrient signaling pathways (5–9). GH deficiency, as well as GH receptor deficiency (GHRD), results in low concentrations of IGF-I and insulin and protects against many aging-associated pathologies, including but not limited to cancer and diabetes, and in lifespan extension in mice (10, 11). A population of humans in rural Ecuador identified with GHRD (also known as Laron syndrome) display no cases of cancer mortality or diabetes, despite being at a higher risk due to the prevalence of obesity (12). Thus, the identification of protein intake as a major modulator of the GH/IGF-I pathway and subsequent activation of the TOR/S6K and RAS/cAMP/PKA signaling cascades provides a powerful intervention to improve long-term health and lifespan from animal models to humans (**Table 1**). The aim of this narrative review is to summarize research on the impact of protein restriction on health and longevity in model organisms and to discuss the ongoing research on the effects of a low-protein fasting-mimicking diet (FMD) on biomarkers of healthy aging in mice and humans.

**TABLE 1:** Definition of terms

Term	Definition
Lifespan	The period of time between the birth and death of an organism
	The average length of life of a kind of organism or of a material object especially in a particular environment or under specified circumstances
Healthspan	The length of time lived in reasonably good health
Longevity	Generally best considered meaning “typical length of life” and sometimes used as a synonym for “life expectancy” in demography
Healthy aging	The process of developing and maintaining the functional ability that enables well-being in older age
Quality of life	The degree to which an individual is healthy, comfortable, and able to participate in or enjoy life events. The term quality of life is ambiguous, because it refers both to the experience of an individual and to the living conditions in which individuals find themselves

## Current Status of Knowledge

### Why protein matters: from single cells to mammals

Yeast (*Saccharomyces cerevisiae*) has been among the first organisms utilized to understand the role of the nutrient-sensing Tor1/serine/threonine-protein kinase in *S. cerevisiae* (Sch9) and Ras2/cAMP/PKA pathways and their role in lifespan through the activation of progrowth and pro-aging signaling (6). In yeast, the deletion of components of this signaling cascade extends chronological lifespan, reduces age-related genome instability, and promotes multistress resistance—a hallmark of healthy aging (6). The activation of Tor1/Sch9 by amino acids and Ras2/cAMP/PKA by glucose results in the inhibition of the regulator of IME2 in *S. cerevisiae* (Rim15), a positive regulator of the multicopy suppressor of SNF1 mutation (Msn2/4) and the Glg1-2 suppressor and transcription factor (Gis1) stress resistance transcription factors (13). Notably, amino acid limitation reduces aging-dependent DNA damage accumulation and increases lifespan through the inhibition of the Tor1/Sch9 cascade (13, 14). Conversely, the amino acids threonine, valine, and serine all promote cellular sensitization and reduce yeast lifespan through the activation of Tor1/Sch9 (14).

In invertebrate models, such as *C. elegans* and *Drosophila melanogaster*, reducing the activity of nutrient-sensing pathways, that is, TOR, through the limitation of amino acids, as well as nitrogen and carbon sources, increases lifespan (1, 15–19). In the fruit fly *D. melanogaster*, the extension of lifespan can be achieved without reducing the calorie content by modulating dietary carbohydrates, yeast (as a protein source), and other macronutrients and their ratios, whereas the supplementation of essential amino acids reverses this lifespan extension; the supplementation of nonessential amino acids has minimal impact on lifespan (20, 21). The protein-to-carbohydrate ratio significantly impacts lifespan: diets high in protein (i.e., yeast extract) and low in carbohydrates (sucrose) have been shown to negatively impact the lifespan of the Queensland fruit fly *Bactrocera tryoni* and *D. melanogaster* (22). Maximal longevity for these flies was observed at a protein:carbohydrate ratio between 10:1 and 20:1 (22). Therefore, protein and/or amino acid intake has major effects on lifespan even in simple model organisms. Next, we discuss how these findings translate to mammals and how protein intake modulates the GH/IGF-I pathway and its downstream effectors.

In rodents, the restriction of protein intake or of specific amino acids, as well as the genetic modulation of the GH/IGF-I pathway, have been shown to increase healthspan, thus emphasizing that protein plays an evolutionarily conserved role in lifespan regulation (1). Restriction of the amino acid tryptophan delays tumor incidence and onset, protects the liver and kidney against ischemia/reperfusion injuries, and lengthens the healthspan (2, 23–25), whereas methionine restriction decreases adiposity and serum glucose, insulin, and IGF-I concentrations, and the mitochondria-dependent production of reactive oxygen species, thereby inducing less oxidative damage in male Wistar rats, male Fisher 344 rats, and female BALB/cJ × C57BL/6J F1 mice (26–29). Although insulin sensitivity is improved following the restriction of leucine in male C57BL/6J mice, which in turn is likely correlated with healthspan, the restriction of this specific amino acid has not been shown to extend lifespan (30, 31). A diet with reduced concentrations of branched-chain amino acids, but isocaloric in total amino acids compared with the control diet, modestly improved glucose tolerance and slowed fat mass gain in healthy male C57BL6 mice (32), but promoted rapid fat mass loss and restored glucose tolerance and insulin sensitivity in obese male C57BL6 mice maintained on a high-fat, high-sugar diet without caloric restriction (CR) (33). Protein restriction with supplementation of nonessential amino acids reduced IGF-I concentrations in male 3xTg-AD mice displaying significant cognitive impairment and Alzheimer disease–like pathology, which resulted in a decrease in tau phosphorylation in the hippocampus, but without affecting the concentrations of β-amyloid, and alleviation of the age-dependent impairment in cognitive performance (34). Here, it should also be mentioned that the healthspan effects observed with CR can be at least partially explained by the restriction of the amino acids methionine and tryptophan that often goes in hand with CR (29, 35, 36). However, in rodents (male and female) specific amino acid restriction can result in food aversion due to a change in taste, which in turn reduces food intake (37, 38). Although this can be controlled for by using pair-wise feeding of control animals in studies focused on protein- or amino acid–restricted diets, the possibility remains that food aversion–induced CR is a confounding variable (20). Protein and carbohydrate, rather than fats, are the predominant macronutrients that modulate food consumption to meet biological requirements (39–41). When testing the lifetime impact of dietary macronutrient ratios—differing in content of protein (5–60%), fat (16–75%), carbohydrate (16–75%), and energy (8, 13, or 17 kJ/g of food)—on metabolic health and longevity in male and female C57BL6 mice consuming ad libitum, low-protein/high-carbohydrate diets resulted in the longest lifespans (39, 40). The mice whose diets included 5–15% protein and 40–60% carbohydrates lived the longest, ≤150 wk compared with 100 wk for those on a diet of ∼50% protein, despite the fact that mice that consumed more protein were leaner (39). The reduction in progrowth signaling of the GH/IGF-I axis in mammals has been extensively studied using genetically engineered mouse models (42). Male and female mice with a GH-insulin/IGF-I signaling deficiency exhibit dwarfism but also increased insulin sensitivity and the delayed manifestation of fatal malignancies and increased health and lifespan (1, 23, 43). Similarly, the inhibition of mammalian target of rapamycin (mTOR)/S6K signaling results in increased lifespan and the reduction of aging phenotypes (7, 44, 45). In summary, essentially all model organisms used in aging research support a link between dietary protein uptake and aging in that reduced protein intake is associated with a sex-independent increase in lifespan.

### Protein intake, health, and longevity in nonhuman primates

As an important first step to demonstrate whether the health benefits observed with CR could translate to humans, the University of Wisconsin (UW) and the National Institute on Aging (NIA) used male and female rhesus monkeys to investigate the impact of a 30% CR on lifespan and healthspan (46, 47). Although the studies resulted in somewhat conflicting data regarding an increase in lifespan (rhesus monkeys in the UW study increased their lifespan whereas there were no effects in the NIA study), both demonstrated healthspan benefits in the CR-fed rhesus monkey cohorts: age-related disease and all-cause mortality were reduced in the CR group (UW) with reduced incidences of cancer in the NIA CR group (46, 47). Thus, whether CR might translate to life extension in humans remains an unanswered but critical question (1, 23, 43). However, one can extrapolate some important results from these studies based on the analysis of the experimental conditions, in particular the food used to feed the animals. Foremost, significantly lower sucrose concentrations were fed in the NIA study (3.95%) compared with the 28.5% in the UW rhesus monkey diet (47). Protein intake in the NIA study was based on wheat, corn, soybean, fish, and alfalfa meal, whereas the UW study used lactalbumin obtained from milk whey as the main protein source, suggesting that the more plant-based protein source diet used in the NIA study might have reduced the risk for aging-related mortality factors compared with the animal-based protein sources used in the UW study (47). Arguably, CR extended lifespan in the UW study because these animals were maintained on unhealthy diets; for the animals fed a more plant-based protein diet at the NIA, CR had no significant effects on lifespan. Thus, in line with the basic animal models described previously, dietary protein content, as well as its source, makes a significant, gender-independent contribution to primate healthspan.

### Protein intake, health, and longevity in humans

What really qualifies as a macronutritionally healthy diet that successfully extends healthy aging remains an unanswered critical question of high priority for human nutrition research. The benefits of reduced protein intake on outcomes of health in humans have been assessed mostly based on clinical trials and epidemiological studies.

#### Clinical studies

Short-term randomized controlled trials, which fail to consider the long-term impact that nutrition really has, often favor the substitution of protein for carbohydrate (i.e., high-protein/low-carbohydrate diets) due to benefits for weight management, blood pressure reduction, and improvements in cardiometabolic biomarkers (such as blood lipid and lipoprotein profiles), and improved glycemic regulation (48–50). Multiple studies have demonstrated that these beneficial health effects largely depend on weight loss, enhanced postprandial satiety, and energy expenditure when exchanging protein for carbohydrate (51). A meta-analysis of 24 trials, including 1063 individuals and a mean ± SD diet duration of 12.1 ± 9.3 wk, indicated that high-protein diets (protein: 30.5 ± 2.4%; carbohydrate: 41.6 ± 3.5%; fat: 27.8 ± 3.2%) produced more favorable changes in body weight (−0.79 kg; 95% CI: −1.50, −0.08 kg), fat mass (−0.87 kg; 95% CI: −1.26, −0.48 kg), and triglycerides (−0.23 mmol/L; 95% CI: −0.33, −0.12 mmol/L) than a standard-protein, low-fat diet (protein: 17.5 ± 1.5%; carbohydrate: 56.9 ± 3.3%; fat: 25.1 ± 3.1%). Changes in concentrations of fasting plasma glucose; fasting insulin; total, LDL, and HDL cholesterol; and blood pressure were similar across dietary treatments (*P* ≥ 0.20) (51). Notably, this meta-analysis included overweight and obese men and women, postmenopausal women, hyperinsulinemic men and women, men and women with metabolic syndrome or with type 2 diabetes, and even a small cohort of men and women with heart failure or polycystic ovary syndrome (women only). Thus, high-protein and low-glycemic diets might improve compliance and maintenance of weight loss in overweight adults (52), yet these diets do not align with the low-protein dietary recommendations that can be considered as generally healthy antiaging diets.

#### Epidemiological studies

Increasing protein intake by 10% (or 5 g of protein) while decreasing carbohydrate intake by 10% (or 20 g carbohydrates) was correlated with a 5% increase in incidences of cardiovascular disease (CVD) in a Swedish study of 43,396 women with an average follow-up of 15.7 y; interestingly, individuals substituted carbohydrates mostly with animal protein, thereby changing their overall protein:carbohydrate intake ratio (53). Multiple other large studies suggest a positive correlation between low-protein diets and lower rates of aging-related disease. In the 26-y follow-up of the Nurses’ Health Study (NHS; including 85,168 women) and a 20-y follow-up of the Health Professionals Follow-Up Study (HPFS; including 44,548 men), diets high in animal-based protein and fats and low in carbohydrates were associated with higher mortality (HR: 1.12; 95% CI: 1.01, 1.24) for both men and women (54). Recent work has indicated that dietary needs change during aging, which should be considered when making recommendations for healthy aging diets. When considering male and female individuals aged ≥50 y in the NHANES dataset, no positive correlation between protein intake and increased mortality was supported (55). However, stratifying this cohort into 2 age groups (50–65 y and >65 y) allowed assessment of nutritional needs and their impact on health based on age cohorts, but the age cohorts were not further divided by sex, which might allow determination of any possible sex-specific differences at older ages. In individuals aged 50–65 y, high protein intake (≥20% protein-derived consumed calories) was associated with a 74% increase in their RR of all-cause mortality (HR: 1.74; 95% CI: 1.02, 2.97), and they were >4 times as likely to die of cancer (HR: 4.33; 95% CI: 1.96, 9.56), an effect not observed in those older than 65 y (55). In fact, individuals aged >65 y who consumed a high-protein diet had a 28% reduction in all-cause mortality (HR: 0.72; 95% CI: 0.55, 0.94) and a 60% reduction in cancer mortality (HR: 0.40; 95% CI: 0.23, 0.71) compared with those consuming low-protein diets, which was not affected when controlling for other fat or carbohydrate intake or the protein source (55). IGF-I concentrations decrease with aging in humans, but in this dataset individuals aged ≥50 y who consumed a high-protein diet also had higher IGF-I concentrations. Elevated circulating IGF-I concentrations are associated with increased risk of developing certain malignancies (56, 57). Thus, it is possible that the benefits of the high-protein intake in those aged ≥65 y rely on maintaining “healthy” IGF-I concentrations (55), which could help to maintain a healthy weight and preserve muscle mass thereby preventing frailty and fall-associated hospitalization (58). This concept is supported by results in mice: young mice can easily maintain a healthy weight on a low-protein diet, whereas older mice struggle to maintain weight and become increasingly frail (55). Hence, although the great majority of studies suggest a negative correlation between high-protein diets and aging-related diseases, the fact that some studies do not support these findings could be due to differences in the age observed and trade-offs in the nutritional needs at various ages.

In Ecuador, a small population of subjects exhibits a rare case of symmetrical dwarfism (Laron syndrome) due to GHRD and characterized by very low (≤20 ng/mL) circulating IGF-I concentrations (12). This population enables further extension of our understanding of the role of the GH/IGF-I axis and its implications for human aging. Longitudinal studies with a 22-y follow-up of this population indicate the absence of aging-related pathologies, such as cancer and diabetes, despite a very high prevalence of overweight and obesity (a risk factor for cancer and diabetes) in male and female subjects (12). Similarly, a different population of 230 subjects with Laron syndrome was protected against cancer (59). Both studies included male and female subjects although the sex distribution is unknown. Male and female GHRD mice show improved age-dependent cognitive performance (60), whereas the effect of GHRD on human cognition remained unexplored until recently. Using MRI, the brain structure, function, and connectivity of 3 male and 10 female subjects with Laron syndrome and 12 unaffected relatives were compared and revealed that the GHRD group displayed enhanced cognitive performance and greater task-related activation in frontal, parietal, and hippocampal regions, results consistent with effects observed in young adults (61).

Dietary patterns in areas with populations with exceptional longevity, so-called “blue zones,” provide further evidence that nutrition in combination with other healthy lifestyle factors (such as exercise, spirituality, and a sense of “belonging”) can have significant impact on human health (62–65). The long-lived populations of Okinawa, Sardinia, Loma Linda, the Nicoya Peninsula, and Ikaria consume predominantly plant-based, low-protein diets that include a high intake of fruits, vegetables, and nuts (66).

In conclusion, the above findings, together with a plethora of additional studies based on human and nonhuman interventions, support the hypothesis that lower protein intake results in lower activity of the GH/IGF-I axis, thereby protecting against the development and onset of aging-related pathologies and aging itself. These effects seem to be also largely sex-independent.

## The Source Matters: Animal- Compared with Plant-Based Protein Intake and Health

### Results from animal models

Historically, experiments designed to determine how different protein sources might affect health and lifespan in model organisms of aging are scarce. This could be related to various reasons such as the typical diet consumed by a specific model organism, which limits what can reasonably be fed to an animal. Another major limitation of the experiments designed to evaluate protein sources is that often only 1 type of animal protein (such as casein) is compared with 1 type of vegetable protein (typically soya); notably this does not reflect the complex composition of most human diets.

Nine-week-old C57BL/6J male mice maintained for 3 wk on either a 20% plant protein–based or a 20% dairy protein–based diet with an equivalent percentage of calories derived from either protein source, indicated that plant protein–based and dairy protein–based diets are indistinguishable with respect to their short-term consequences on weight, body composition, and control of glucose homeostasis as assessed by glucose, insulin, and pyruvate tolerance tests in mice (67). Male rabbits fed with animal protein, but not soya protein, developed vascular lesions and displayed increased atherosclerosis and plaque formation that was independent of dietary cholesterol and saturated fat (68, 69). In fact, casein was 5-fold more atherogenic than soya protein over just a 6-mo feeding period (70), in part because casein intake increases cholesterol concentrations whereas soya protein decreases cholesterol concentrations in the serum (71, 72). Additionally, cow milk–derived lactalbumin increased atherosclerosis >2-fold over corn- or wheat-derived protein (73). Animal protein from 12 different sources elevated cholesterol concentrations compared with 11 kinds of plant-derived protein, thus making it likely that these effects translate to other animal- and plant-based proteins (70). Although the referenced studies in this section rely largely on data from male mice and rabbits, this does not exclude that the same adverse metabolic health effects of animal-based protein sources likely persist in females. In fact, we commented above on the effects that different dietary composition could have had on the healthspan in male and female rhesus monkeys maintained on a CR diet (47).

### Protein source and health outcomes in humans

As outlined above, the amount of protein consumed impacts aging and health. However, as omnivores, humans do not consume only 1 sort of protein, and hence, which protein source we choose will inevitably influence other components of our diet, such as macronutrients, micronutrients, and phytochemicals, all of which individually and in combination can in turn influence health outcomes. Therefore, we must determine the effects that the source material can have to better understand the long-term health effects of protein intake and optimize dietary recommendations. However, very few studies have considered this, which limits the conclusions that can be drawn regarding protein sources and their relation to all-cause mortality or cause-specific mortality.

For humans, dietary regimens, including dietary composition and diet-associated practices, form the largest group of risk factors for disability and mortality caused by chronic diseases (74). Prospective and randomized clinical trials demonstrate that in humans low-protein diets enhance metabolic health, promote lean physical appearance, lower blood glucose, and decrease the risk of developing diabetes (1, 75). In a cohort of 29,017 postmenopausal women without previous diagnosis of cancer, coronary artery disease (CAD), or diabetes, nutrient density models based on mailed questionnaires were used to estimate risk ratios from a simulated substitution of total and type of dietary protein (76). For women in the highest intake quintile, CAD mortality decreased by 30% (95% CI: 0.49, 0.99) from an isocaloric substitution of vegetable for animal protein. CAD mortality was associated with red meats (risk ratio: 1.44; 95% CI: 1.06, 1.94) and dairy products (risk ratio: 1.41; 95% CI: 1.07, 1.86) (76). Although no significant correlation between overall protein intake levels and ischemic heart disease (IHD) (RR: 0.97; 95% CI: 0.75, 1.24) or stroke events (RR: 1.02; 95% CI: 0.84, 1.24) was measurable among 43,960 middle-aged (53 ± 10 y) men during an 18-y follow-up, comparison of protein source groups provided further insight into the effects of animal-based compared with plant-based protein: an inverse correlation between plant-based protein intake and IHD (RR: 0.64; 95% CI: 0.48, 0.86) or stroke incidence (RR: 0.72; 95% CI: 0.57, 0.90) in the top compared with the bottom quintile, as well as a negative correlation between animal-based protein intake and IHD and stroke has been demonstrated (77, 78). In the NHS and HPFS cohorts, high animal-based protein consumption was associated with higher all-cause mortality (HR: 1.23; 95% CI: 1.11, 1.37), cardiovascular mortality (HR: 1.14; 95% CI: 1.01, 1.29), and cancer mortality (HR: 1.28; 95% CI: 1.02, 1.60). In contrast, vegetable-based protein intake was associated with lower all-cause mortality (HR: 0.80; 95% CI: 0.75, 0.85) and cardiovascular mortality (HR: 0.77; 95% CI: 0.68, 0.87) for both men and women (54). Analyses of the NHS cohort of 84,136 women aged 30–55 y, with no known cancer, diabetes mellitus, angina, myocardial infarction, stroke, or other cardiovascular disease, showed that higher intakes of red meat (RR: 1.16; 95% CI: 1.09, 1.23), red meat excluding processed meat (RR: 1.19; 95% CI: 1.07, 1.32), and high-fat dairy (RR: 1.03; 95% CI: 1.0, 1.06) were significantly associated with elevated risk of CAD. Vegetable protein was significantly associated with an 18% decreased risk when comparing the lowest and the highest intakes across quintiles (RR across quintiles: 1.00, 0.88, 0.85, 0.80, 0.72). Higher intakes of poultry (RR: 0.9; 95% CI: 0.75, 1.08), fish (RR: 0.81; 95% CI: 0.66, 1.0), and nuts (RR: 0.78; 95% CI: 0.66, 0.93) were significantly associated with lower risk of CAD (79). Using the NHANES dataset, animal protein was positively associated with all-cause mortality (55). Yet, before drawing any conclusions, noteworthy limitations of these studies have to be considered, which can include relatively small sample sizes, the form of dietary assessments, and the evidently scarce data on animal and plant protein sources. Further complicating the issue is that consuming plant-based vegetarian or vegan diets can also be associated with consuming less overall dietary protein and reduced levels of the essential amino acid methionine, both of which diet patterns promote health and longevity in rodents (27, 80, 81). Some of these concerns were largely addressed in a study that utilized data from 2 large US cohort studies with >130,000 participants, repeated measures of diet, and ≤32 y of follow-up to compare animal and plant protein and the risk of all-cause and cause-specific mortality (82). A study population from the NIH American Association of Retired Persons (AARP) Diet and Health Study cohort (83) of half a million people aged 50–71 y at baseline, with a 10-y follow-up, further supports these findings: men and women in the highest compared with the lowest quintile of red meat intakes had elevated risks for overall mortality (men, HR: 1.31; 95% CI: 1.27, 1.35, and women, HR: 1.36; 95% CI: 1.30, 1.43), CVD (men, HR: 1.27; 95% CI: 1.20, 1.35, and women, HR: 1.50; 95% CI: 1.37, 1.65), and cancer mortality (men, HR: 1.22; 95% CI: 1.16, 1.29, and women, HR: 1.20; 95% CI: 1.12, 1.30). In contrast, high intake of white meat and a low-risk meat diet were associated with a small decrease in total and cancer mortality (83). Additional studies further establish a positive correlation between red meat and high-fat dairy consumption and risks for developing age-related diseases, including cancer and diabetes (84, 85). These findings also suggest that processing of meat products might play a significant role in promoting adverse health outcomes. Indeed, well-done red meat; frequent frying, barbecuing, and broiling of meats; and the processing-induced exposure to bioavailable carcinogens, such as heterocyclic aromatic amines, are positively associated with the development of several cancers (86–88). Similarly to what has been shown for red meat alone, the consumption of processed red meat increases the risks for overall mortality (men, HR: 1.16; 95% CI: 1.12, 1.20, and women, HR: 1.25; 95% CI: 1.20, 1.31), CVD (men, HR: 1.09; 95% CI: 1.03, 1.15, and women, HR: 1.38; 95% CI: 1.26, 1.51), and cancer mortality (men, HR: 1.12; 95% CI: 1.06, 1.19, and women, HR: 1.11; 95% CI: 1.04, 1.19) when compared with the lowest intake quintile (83).

The intestinal microbiome plays an important role in modulating the risk of several chronic diseases such as obesity, type 2 diabetes, CVD, and cancer (89). Long-term animal- or plant-based nutrition influences the structure and activity of the micro-organisms residing in the human gastrointestinal system. The short-term consumption of diets composed entirely of animal or plant products rapidly (1–2 d) altered microbial community structure in 6 male and 4 female US volunteers aged 21–33 y, whose BMIs ranged from 19 to 32 kg/m^2^: an animal-based diet increased the abundance of bile-tolerant micro-organisms (*Alistipes, Bilophila*, and *Bacteroides*) and decreased the abundance of *Firmicutes* bacteria that metabolize dietary plant polysaccharides; increases in the abundance and activity of *Bilophila wadsworthia* support a link between dietary fat, bile acids, and micro-organisms associated with inflammatory bowel disease (90).

Thus, long-term adherence to high-protein diets, particularly in combination with indiscrimination toward protein sources, has been linked to adverse health consequences in men and women. Conversely, plant-based protein consumption is associated with a reduced risk for multiple diseases and overall mortality. These results complement the recommendations by the American Institute for Cancer Research and the World Cancer Research Fund to reduce red and processed meat intake to decrease cancer incidence. Therefore, a moderate consumption of plant-based protein should be considered to cover the body's nutritional requirements.

## Health Outcomes of the FMD in Mice and Humans

### Fasting is potent but likely not feasible for most subjects

Poor nutritional habits accumulate and contribute to the time-dependent decrease in metabolic fitness and physiological function associated with increasing age. However, correcting unhealthy dietary habits by interventions such as CR, low-protein diets, and specific variants of fasting (such as time-restricted feeding, intermittent fasting, and alternate-day fasting) have been proven to at least partially delay biological aging and restore physiologically normal function (1, 2, 23, 43, 91). Similarly to the results obtained in many model organisms, fasting in humans can be utilized to prevent and treat diseases. Water-only fasting for 10–14 d, followed by a low-fat, low-sodium vegan-based refeeding period for ≤6–7 d, reduced systolic blood pressure in 174 hypertensive patients (sex not specified) by 37/13 mm Hg, a >2-fold change compared to the 17/13 mm Hg reduction following an intervention comprising 12 d of exercise and a vegan low-fat, low-salt diet in 500 male and female subjects with various health problems including hypertension and CAD (92, 93). In a large cohort with >2000 participants (80.3% female, 19.7% male) with chronic diseases, a very-low-calorie diet of 350 kcal/d was considered safe, demonstrating that such interventions can be successfully applied under medical supervision even to ill subjects (94). In 107 overweight or obese premenopausal women, both chronic CR- and intermittent-fasting–based interventions were equally efficient in reducing weight loss and biomarkers associated with weight loss and metabolic disease risk (95). A caveat to almost all of these diets is that they require major lifestyle changes and continuous implementation in daily living routines. It is highly arguable if chronic interventions can be successfully integrated after a lifetime of unhealthy food choices, whether those choices are because of cost or other socioeconomic factors or convenience, despite increasing awareness and education focused on healthy foods (i.e., “food pyramids” in the United States). In line with the arguments above, clinical trials based on CR and other dietary interventions often experience drop-out rates of ∼15–40% (96, 97), emphasizing that even health-cautious and/or motivated volunteers are unable to adhere to these long-term interventions (98, 99).

### FMD increases health and longevity in mice

To overcome the fact that many fasting-based interventions are likely not feasible or extremely difficult to adhere to, we developed a periodic, short-term, low-calorie, and low-protein dietary intervention in line with the health advantages of low-calorie or low-protein diets described above. The short duration (5 consecutive days per month recommended for humans) together with the periodic application is thought to improve adherence and reduce dietary “fatigue,” thus enabling easy inclusion into existing lifestyles.

We tested the efficacy of this FMD in multiple mouse studies focused on health and longevity (100), for the treatment of aging-related diseases such as diabetes (101), cancer (102), and in a mouse model for multiple sclerosis (103). In a first effort, we tested bimonthly cycles of this FMD in middle-aged female C57BL/6 mice and observed a decrease in the size of multiple organs/systems, which was followed by an elevated number of progenitor and stem cells and tissue regeneration upon refeeding (100). Similarly to the effects of 72 h of short-term starvation in mice, the FMD induced a 45% reduction in IGF-I concentrations compared with baseline values (100). Almost lifelong feeding of the FMD extended longevity, lowered visceral fat, reduced cancer incidence and skin lesions, rejuvenated the immune system, and retarded bone mineral density loss. FMD cycles promoted hippocampal neurogenesis and elevated NeuroD1, which likely explains the improved cognitive performance observed in the FMD-fed mice when compared with mice maintained on a standard rodent diet. In type 2 and type 1 diabetes mouse models, FMD cycles successfully restored insulin secretion and glucose homeostasis following the generation of insulin-producing β-cells, with a gene expression profile resembling that observed during pancreatic development (101). In cancer treatment, the combination of the FMD with chemotherapy in mice delayed the progression of breast cancer and melanoma by increasing the number of bone marrow common lymphoid progenitor cells and cytotoxic CD8+ tumor-infiltrating lymphocytes and, at least in a mouse breast cancer model, the downregulation of the stress-responsive enzyme heme oxygenase-1 (102). In an experimental autoimmune encephalomyelitis mouse model, 3-d cycles of the FMD were associated with increased corticosterone concentrations and regulatory T-cells; reduced concentrations of proinflammatory cytokines, TH1 and TH17 cells, and antigen-presenting cells; and amelioration of demyelination, all of which likely resulted in the observed reduced clinical severity; with completely reversed symptoms in 20% of the mice (103). These preclinical results indicate that the FMD provides a potent intervention to extend healthy aging as well as powerful treatment modality that warrants further investigation in human trials.

### FMD reduces risk factors for multiple diseases in humans

Although the number of trials using FMDs with various diseases is expanding, only limited disease-specific findings in humans are currently available. In ex vivo experiments using primary human pancreatic islets derived from type 1 diabetic subjects, fasting-like conditions (low glucose and IGF-I) reduced PKA and mTOR activity and induced insulin production similar to the effects seen in diabetic mice (101). IGF-I treatment reversed the FMD-induced health benefits in this type 1 diabetes model (101).

In a pilot clinical trial in 19 healthy volunteers (7 women and 12 men), 3 monthly FMD cycles (5 d per cycle) decreased risk factors/biomarkers such as body weight, serum glucose, IGF-I, trunk fat, and others associated with aging, diabetes, CVD, and cancer without major adverse effects (100). Notably, mesenchymal stem and progenitor cells in the peripheral blood mononucleated cell population showed a trend to increase at the end of FMD, in line with the results obtained in mice (100). The results on markers/risk factors associated with aging and age-related diseases and the FMD-induced improvements were confirmed in a randomized crossover-style clinical trial (104). Generally healthy participants (63% female and 37% male) from the United States consuming a nonsupervised unrestricted diet were compared with volunteers who consumed the FMD for 5 consecutive days per month for 3 mo. Whereas the FMD reduced body weight and trunk and total body fat, lowered blood pressure, and decreased IGF-I in all subjects who completed the trial, a post hoc analysis demonstrated that BMI, blood pressure, fasting glucose, IGF-I, triglycerides, total and LDL cholesterol, and C-reactive protein were more beneficially affected in participants at risk of disease than in subjects who were not at risk (104). Thus, cycles of a 5-d FMD are safe, feasible, and effective in reducing markers/risk factors for aging and age-related diseases and might be potentially effective in the treatment of relapsing remitting multiple sclerosis patients (*n* = 18; 83% female and 17% male) (103). The FMD studies are currently expanding to include patients with diagnosed diseases to confirm the effect of the FMD on disease prevention and treatment. Together, the findings indicate that FMDs might present a potent dietary intervention that can be utilized alone, or in combination with existing treatment strategies such as chemotherapy-based cancer treatment. However, as mentioned before, the number of clinical studies using FMDs is still small and thus any approach to include available FMDs into any treatment has to be carefully evaluated and supervised by a trained clinician.

## Conclusions

**FIGURE 1:**
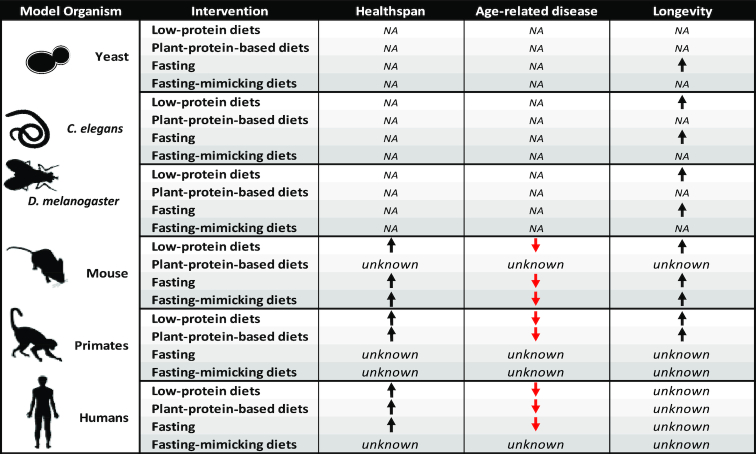
Effects of low-protein-based and plant-protein-based diets, fasting, and fasting-mimicking diets.

Investigations of the amount of protein intake as well as the protein source in a variety of model organisms, nonhuman primates, and in humans emphasize a significant role for dietary protein in health and longevity (**Figure 1**). Although we acknowledge that more research is needed, the existing literature, covering basic preclinical, randomized, and epidemiological experiments with large datasets, supports that protein intake and the resulting activation of nutrient-sensing pathways regulate metabolism, growth, and aging. All available evidence demonstrates that low protein consumption, specifically based on plant-derived sources, is associated with benefits for healthy aging, at least until the age of 65. Further research is needed to establish how nutrient requirements change throughout the lifespan, and to identify the potential role of sex-dependent differences in nutrient response and needs, particularly at advanced ages when system integrity becomes increasingly frail. Today, dietary interventions remain the most applicable and cost-efficient means of preventing and treating a wide variety of aging-related diseases for most humans. One of these interventions, the FMD, has gained significant attraction over the past year due to its safety and feasibility, easy inclusion into personal lifestyle choices, and most of all its high efficacy in reducing risk factors associated with aging, diabetes, CVD, and cancer. However, clinical studies using FMDs are limited and thus require careful evaluation.

## Notes

Published in a supplement to *Advances in Nutrition*. This supplement was sponsored by the Harding-Buller Foundation of Ohio. The contents are solely the responsibility of the authors and do not necessarily represent the official views of the sponsors. Publication costs for this supplement were defrayed in part by the payment of page charges. The opinions expressed in this publication are those of the authors and are not attributable to the sponsors or the publisher, Editor, or Editorial Board of *Advances in Nutrition*.

SB is supported by the Irene Diamond Fund/AFAR Postdoctoral Transition Awards in Aging. VDL is supported by National Institutes of Aging grant AG055369; the Department of Defense grant 12291270; the William H Donner Foundation; the Glenn Award for Research in Biological Mechanisms of Aging; and the Create Cures Foundation.

Author disclosures: SB report no conflicts of interest. VDL has equity interest in L-Nutra, a company that develops medical food. The University of Southern California has licensed intellectual property to L-Nutra. As part of this license agreement, the University has the potential to receive royalty payments from L-Nutra.

Abbreviations used: CAD, coronary artery disease; CR, caloric restriction; CVD, cardiovascular disease; FMD, fasting-mimicking diet; GH, growth hormone; GHRD, growth hormone receptor deficiency; HPFS, Health Professionals Follow-Up Study; IGF-I, insulin-like growth factor I; IHD, ischemic heart disease; mTOR, mammalian target of rapamycin; NHS, Nurses' Health Study; NIA, National Institute on Aging; PKA, protein kinase A; Sch9, serine/threonine-protein kinase in *S. cerevisiae*; S6K, ribosomal protein S6 kinase; TOR, target of rapamycin; UW, University of Wisconsin.
